# Tailoring PLA/ABS Blends Compatibilized with SEBS-g-MA through Annealing Heat Treatment

**DOI:** 10.3390/polym15163434

**Published:** 2023-08-17

**Authors:** Anna Raffaela de Matos Costa, Carlos Bruno Barreto Luna, Emanuel Pereira do Nascimento, Eduardo da Silva Barbosa Ferreira, Claudia de Matos Costa, Yeda Medeiros Bastos de Almeida, Edcleide Maria Araújo

**Affiliations:** 1 Academic Unit of Materials Engineering, Polymer Processing Laboratory, Federal University of Campina Grande, Av. Aprígio Veloso, 882, Bodocongó, Campina Grande 58429-900, PB, Brazil; raffaela_matos@yahoo.com.br (A.R.d.M.C.); emanueluepb@gmail.com (E.P.d.N.); eduardosbf95@gmail.com (E.d.S.B.F.); edcleidemaraujo@gmail.com (E.M.A.); 2 Academic Unit of Mechanical Engineering, Federal University of Campina Grande, Av. Aprígio Veloso, 882, Bodocongó, Campina Grande 58429-900, PB, Brazil; claudiamatos1@yahoo.com.br; 3 Department of Chemical Engineering, Federal University of Pernambuco, Recife 50740-520, PE, Brazil; yeda.oliveira@ufpe.br

**Keywords:** poly (lactic acid), compatibilizing, annealing, impact strength, SEBS-g-MA

## Abstract

In this work, blends based on poly (lactic acid) (PLA)/acrylonitrile-butadiene-styrene (ABS) compatibilized with maleic anhydride-grafted (SEBS-g-MA) were prepared in a co-rotational twin-screw extruder by varying the concentrations of the compatibilizing agent. The influence of the compatibilizing agent on the morphology, mechanical, thermal, thermomechanical, and rheological properties of the prepared materials was analyzed. The effect of annealing on the properties of the blends was also investigated using injection-molded samples. The X-ray diffraction (XRD) results proved that the increments in crystallinity were an effect of annealing in the PLA/ABS/SEBS-g-MA blends, resonating at higher heat deflection temperatures (HDTs). The impact strength of the PLA/ABS blends compatibilized with 10 wt% SEBS-g-MA was significantly increased when compared to the PLA/ABS blends. However, the hardness and elastic modulus of the blends decreased when compared to neat PLA. The refined morphology shown in the scanning electron microscopy (SEM) analyses corroborated the improved impact strength promoted by SEBS-g-MA. The torque rheometer degradation study also supported the increased compatibility between SEBS-g-MA, PLA, and ABS. The TGA results show that the PLA/ABS and PLA/ABS/SEBS-g-MA blends are more thermally stable than the neat PLA polymer at higher temperatures. The results showed that the ideal composition is the heat-treated PLA/ABS/SEBS-g-MA (60/30/10 wt%), given the high impact strength and HDT results. The results of this work in terms of mechanical improvement with the use of compatibilizers and annealing suggest that the PLA/ABS/SEBS-g-MA system can be used in the production of 3D-printing filaments.

## 1. Introduction

Poly (lactic acid) (PLA) is one of the biopolymers that has attracted the most interest due to its mechanical properties, such as tensile strength, flexural strength, and elastic modulus, which are superior to those of conventional polymers, including PS, PP, and PE, and are comparable to those of PET [1]. These characteristics and the biodegradability and biocompatibility of PLA have led to its use in many applications, especially in agricultural films, degradable food packaging, and the healthcare sector. However, PLA is very brittle, with less than 10% elongation at break, which limits its use in applications requiring plastic deformation under high stress [2]. Therefore, many methods, such as copolymerization or blending with other polymeric materials, have been studied and developed to overcome the limitations of PLA, especially the stiffness-hardness [3,4,5,6,7].

The acrylonitrile-butadiene-styrene (ABS) copolymer can be an impact modifier for toughening aliphatic polyesters. ABS exhibits dimensional stability and a high toughening potential since the ABS particles behave like rubber, acting as an impact strength modifier [8]. ABS is a thermoplastic usually consisting of polybutadiene (PB) particles dispersed in a styrene-acrylonitrile (SAN) matrix. It is widely used as an impact modifier for polycarbonate [9] and polyamide (PA) [10], given its excellent toughness and low cost compared to other toughening resins, such as EPDM. ABS was also reported as a good candidate to strengthen PLA [11,12,13,14,15].

However, PLA/ABS blends are immiscible and incompatible, exhibiting undesirable mechanical properties, such as low tensile strength and low impact strength, caused by poor interactions between the components [13,16,17]. Li and Shimizu [18] used ABS to harden PLA. The mechanical results showed that all PLA/ABS blends have higher flexural strength and lower elastic modulus than neat PLA. An alternative to minimize resin incompatibility is introducing a third component that acts as an interfacial agent called a compatibilizing agent [18,19]. Rigoussen et al. (2019) [20] investigated blend compatibilizers based on crude and epoxidized cardanol by reacting them with PLA or ABS. They observed outstanding improvements in impact strength (+172% when compared to neat PLA) and elongation at break (+1000% compared to neat PLA).

In order to achieve the compatibilization of PLA and ABS, a compound, such as SEBS-g-MA, is added during the melting process [21,22,23]. In fact, SEBS can be grafted onto ABS, leading to a better interaction at the interface between the two polymers. Adding maleic anhydride to the SEBS structure improves the reactivity between the compatibilizer and PLA during melt processing. The literature shows that maleic anhydride groups are reactive with the end of the PLA chain. Indeed, the maleic anhydride groups are known to be reactive with the chain end of PLA [21,22,23].

Some works in the literature have already shown the efficiency of SEBS-g-MA as a compatibilizer. Essabir et al. (2020) [24] reported an increase in the storage modulus, loss modulus, and the complex viscosity of PA6/ABS blends compatibilized with SEBS-g-MA when compared to pristine blends. Sangeetha et al. (2016) [25] used SEBS and maleic anhydride-grafted SEBS (SEBS-g-MA) in PLA and concluded that the PLA/SEBS-g-MA blends had a significant increase in elongation at break and impact strength when compared to PLA and PLA/SEBS blends.

Polymer processing almost always causes internal stresses in products. Parts with high molding stresses may exhibit problems when in service, including brittleness, warping, and low resistance to stress cracking [26]. For such cases, an annealing procedure may be necessary. PLA is one of the materials that undergo more transformations with secondary crystallization and, thus, is more sensitive to the effects of annealing and has a slower crystallization rate [27]. Annealing allows its molecular chains to move toward a more semi-crystalline structure, thus achieving better mechanical properties. Annealing increases tensile strength and creep resistance by increasing the percentage of crystallinity, reducing air gaps, improving layer-to-layer adhesion, and removing internal stresses [28]. Studies [29] show that annealed PLA parts gain 10 to 20% in strength with the bonus of becoming less brittle. Concerning temperature resistance, annealed PLA also shows superior results to other 3D-printed annealed materials. Jayanth et al. 2022 [30] evaluated the effect of annealing on 3D filaments using PLA. The tensile properties were increased by about 80% after heat treatment at about 100 °C for 4 h. The HDT test also showed that the heat resistance of the heat-treated parts increased by about 73% when compared to the non-heat-treated ones. Hence, two approaches were investigated in this work:

- The first approach was to examine the effect of SEBS-g-MA, assuming that the styrene blocks of SEBS are miscible with the styrene groups of ABS [24]. In contrast, the maleic anhydride groups would react with the hydroxyl groups of PLA [26,31], which should be preferentially located at the interface to act as an effective compatibilizer;
- In the second approach, we examined the effect of annealing heat treatment in relieving internal stresses and preventing secondary crystallization.

## 2. Materials

The polymer used was poly (lactic acid) (PLA) in a granular form supplied by 3DLAB from NatureWorks^®^ Ingeo™ 2003D (Plymouth, MN, USA). It has a melt flow index of 6 g/10 min and a density of 1.24 g/cm^3^. Acrylonitrile-butadiene-styrene terpolymer (ABS) was in pellet form, supplied by Innova (São Paulo, Brazil): commercial code AE8000^®^, with a density of 1.04 g/cm^3^ and a melt flow index of 5 g/10 min (220 C/10 kg). The ABS employed had the following monomeric mass proportion: styrene monomer: between 55% and 85%, acrylonitrile monomer: from 20% to 27%, and butadiene monomer: from 12% to 20%. The compatibilizer used was styrene-(ethylene-butylene)-styrene copolymer (SEBS-g-MA), commercialized with the code FG1901, containing 1.7% maleic anhydride, with a density of 0.91 g/cm^3^. SEBS-g-MA was used in pellet form and was supplied by Kraton (São Paulo, Brazil).

### Blend Processing

PLA, ABS, and their blends, with and without compatibilizer, were processed in a modular corotational twin-screw extruder, model Zweiwelliger Schneckenkneter (D = 18 mm and L/D = 40) from Coperion Werner & Pfleiderer (Stuttgart-Feuerbach, Germany), at 250 rpm under the following temperature profile: 180; 190; 200; 200; 210; 210 °C. Before processing, the materials were dried in a vacuum oven for 24 h at 60 °C. Table 1 presents the compositions of the PLA/ABS blends containing different concentrations of the SEBS-g-MA copolymer.

The PLA/ABS and PLA/ABS/SEBS-g-MA systems were molded in an Arburg Allrounder Injector 207C Golden Edition (Radevormwald, Germany) injection molding machine, using an injection temperature profile of 180 °C, 190 °C; 190 °C; 200 °C; 210 °C. The cooling time was 30 s, and the mold temperature was 20 ºC. The tensile, impact, and HDT specimens were molded according to the ASTM D638, D 256, and ASTM D648 standards. Annealing was performed on the injection specimens kept in a vacuum oven for 3 h at 90 °C; these parameters were obtained from previous studies [26].

## 3. Methodology

### 3.1. Rheological Characterization

Rheological characterization was performed in a Scientific Haake Torque Rheometer PolyLab QC Mixer (Thermo Fisher Scientific, Waltham, MA, USA), at 60 rpm and 200 °C for 15 min, wherein the materials were added simultaneously into the mixing chamber with a fill factor of 70%. The degradation rate and rheological parameters of the polymers and blends containing 60% PLA and 40% ABS with different concentrations of SEBS-g-MA were estimated by analyzing the torque and temperature at the final stages of processing, as has already been tested and documented in several papers in the literature. The analysis of T(t) and Z(t) provided by the internal mixer during the last processing stage—melt processing—allows for an estimate of the rheological characteristics (dependence of viscosity on temperature and shear rate) of the processed material and an evaluation of the incipient degradation rate during processing, as has already been extensively tested in several works [32,33,34,35,36]. The relative rate change of the terminal adjusted torque is given by

(1) $$R_{Z}=\frac{1}{\overset{¯}{Z^{*}}}\frac{dZ^{*}}{dt}.$$

where Z* is the adjusted torque, calculated according to the literature, and *R_Z_* is a measure of the degradation rate (*R_Z_* < 1) or chain extension (*R_Z_* > 1). The value 100 *R_Z_* is the % change in adjusted torque per unit of time under terminal processing conditions. The data analysis methodology employed is discussed in detail by Canedo et al. (2017) [37].

### 3.2. Mechanical Characterization

Izod impact strength tests were performed on notched specimens (ASTM D256) using a Ceast Resil 5.5 J (Turin, Italy), operating with a 2.75 J hammer at room temperature (20 °C). The results were analyzed by averaging 10 specimens. 

The tensile test was performed according to ASTM D 638 in an EMIC DL 2000 mechanical testing machine (São Paulo, Brazil), with a loading speed of 5 mm/min and a load cell of 20 kN. The testing was conducted at room temperature, and the average results of the 10 specimens were reported.

The Shore D hardness test was carried out according to ASTM D2240, using Metrotokyo (São Paulo, Brazil) equipment under a 50 N load and 10 s of stabilization. The reported results were an average of 10 penetrations.

### 3.3. Thermomechanical Properties

Heat deflection temperature (HDT) was recorded following the ASTM D648 standard, applying a load of 1.82 MPa, a heating rate of 120 °C/h, and a deflection of 0.25 mm. A Ceast HDT 6 VICAT (Turin, Italy) was used to obtain the HDT of three specimens.

### 3.4. Differential Scanning Calorimetry (DSC)

The differential scanning calorimetry (DSC) test was performed in a METTLER TOLEDO DSC (Columbus, OH, USA), using an aluminum sample holder heated from room temperature to 250 °C at a heating rate of 10 °C/min. A nitrogen atmosphere with a flow rate of 50 mL/min was employed throughout the analyses.

### 3.5. Thermogravimetric Analysis (TGA)

The thermogravimetric analyses were performed in Mettler Toledo apparatus (Columbus, OH, USA) at a heating rate of 10 °C/min, with the temperature ranging from ambient to 600 °C in an air atmosphere flowing at 50 mL/min.

### 3.6. Scanning Electron Microscopy (SEM)

Scanning electron microscopy (SEM) analysis was carried out on the fractured surface of the impact specimens. An SSX 550 Superscan-Shimadzu (Kyoto, Japan) was used as the scanning electron microscope, with a voltage of 30 kV under a high vacuum. The fractured surfaces of the specimens were subjected to gold sputtering before SEM analyses. 

### 3.7. X-ray Diffraction (XRD)

X-ray diffraction was performed in a Bruker D2 Phaser (Thermo Fisher Scientific, Waltham, MA, USA) diffractometer operated at a voltage of 30 kV, a current of 30 mA, and a radiation source of CuKα (λ = 1.542 Å). A continuous scan with 2θ between 5° and 35° was performed at a speed of 2°/min and a step of 0.02°.

### 3.8. Fourier Transform Infrared Spectroscopy (FTIR)

FTIR analyses were performed on a PerkinElmer spectrometer (model Spectrum 400 FT-IR/FT-NIR) (Thermo Fisher Scientific, Waltham, MA, USA), using an ATR accessory, scanning from 4000 to 650 cm^−1^ with a resolution of 4 cm^−1^. Then, using the Unscrambler software (version 9.7), the main component analysis (PCA) was performed, with the data centered on the mean and cross-validation to classify the samples.

## 4. Results and Discussions

### 4.1. Degradation during Processing

**Figure 1:**
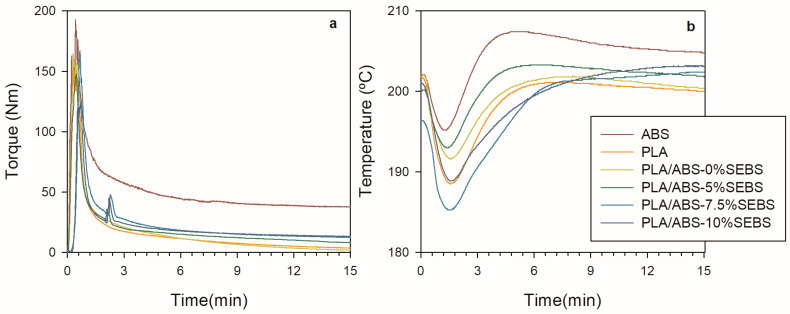
Torque (**a**) and temperature (**b**) as a function of time for PLA and PLA/ABS processing with different SEBS-g-MA contents.

The raw data of temperature and torque vs. time during the processing of the PLA/ABS blends with different concentrations of the SEBS-g-MA compatibilizer studied in this work are shown in Figure 1. The polymers were substantially melted after 4–5 min of processing time. In preparing the PLA/ABS/SEBS-MA blends, SEBS-g-MA was added to the mixing chamber after 2 min, with PLA/ABS already being molten.

Torque depends on polymer viscosity and, consequently, on temperature. The concept of adjusted torque is used here to eliminate torque dependence on the temperature and leave only its variation with time. A detailed description of the fundamentals of this concept can be found in other works [38]. The torque was adjusted at the reference temperature T* = 200 °C, using *β* = 0.02 °C − 1 according to Equation (2) below.

(2) $$Z^{*}=Z\exp\left\{+\beta \left(T-T^{*}\right)\right\}.$$

**Figure 2:**
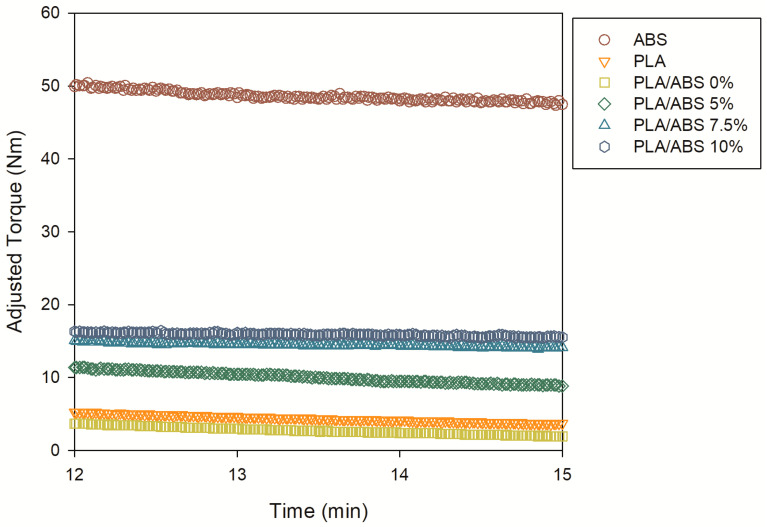
Adjusted torque at 200 °C as a function of time for the processing of the PLA and PLA/ABS blend in the 12–15 min range.

Figure 2 shows the results of the last two minutes of processing (8–10 min).

**Figure 3:**
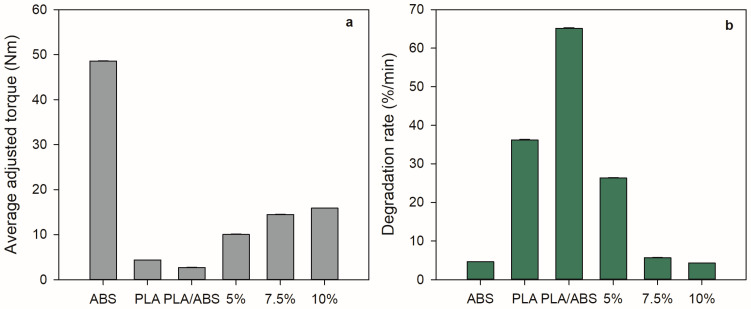
Average adjusted torque (**a**) and degradation rate (**b**) for all samples tested.

The highest torque was observed for ABS. All blends showed a slight decrease in torque with time, suggesting that the blends present higher degradation rates due to the presence of PLA. The torque reduction in the final stages of processing indicates chain scission, which results in lower viscosity and therefore allows for a mixing process with less energy. The average adjusted torque (Figure 3a) and the relative degradation rate (Figure 3b) have been calculated to quantify these trends. The relative degradation rate was estimated using Equation (1). Table 2 presents the numerical results.

Figure 3a shows the average values of the adjusted torque for all systems investigated. Due to the immiscibility between ABS and PLA, the PLA/ABS blend had the lowest torque value when compared to the neat polymers and the compatibilized blends. The adjusted torque is sensitive to SEBS-g-MA content. With the addition of SEBS-g-MA, torque increases significantly when compared to the neat blends. Consequently, the blends compatibilized with SEBS-g-MA showed higher viscosity, suggesting better compatibility between the PLA and ABS components.

Figure 3b confirms the adjusted torque trend since a significant decrease in the degradation rate is observed with increasing SEBS-g-MA content in the PLA/ABS/SEBS-g-MA blends. At the same time, the effect was more prominent for 10 wt% SEBS-g-MA, indicating that the blends compatibilized with 10 wt% SEBS-g-MA degraded much less than neat PLA and ABS, which is in agreement with previous experience [14]. The dependence of the matrix degradation rate on the compatibilization content was significant, suggesting higher resistance to thermomechanical degradation. The higher resistance to thermomechanical degradation may result from the improved compatibility between PLA and ABS due to possible interactions between the styrene blocks in SEBS-g-MA and ABS and of maleic anhydride (MA) with the hydroxyl groups of PLA.

### 4.2. X-ray Diffraction (XRD)

**Figure 4:**
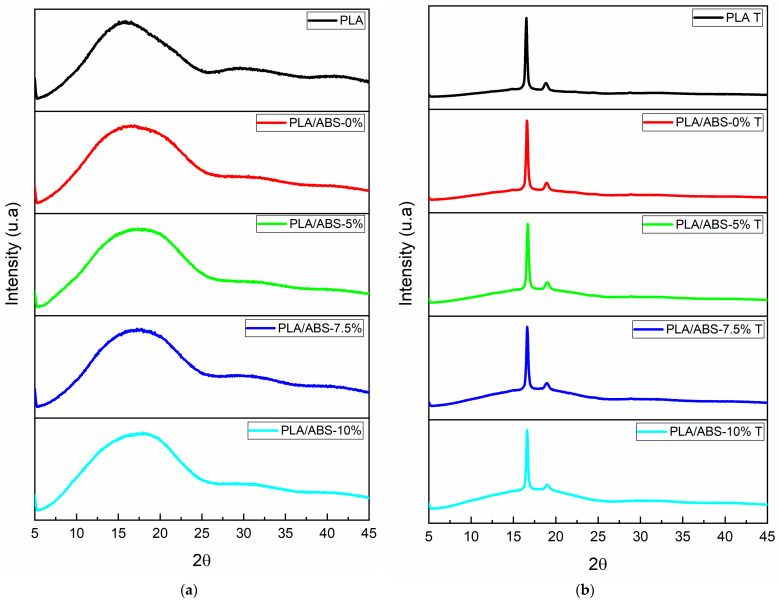
Diffractograms for neat PLA and the PLA/ABS blends with 5, 7.5, and 10% SEBS before (**a**) and after annealing treatment (**b**).

XRD analysis was performed to evaluate the effect of the thermal treatment on the degree of crystallinity. Figure 4 shows the data collected before and after annealing. Depending on the preparation conditions, PLA crystallizes in three different crystalline structures (α, β, and γ forms) [39]. Before annealing, the neat PLA and the PLA/ABS and PLA/ABS/SEBS blends showed the broad band characteristic of an amorphous material [26]. After heat treatment, The PLA, PLA/ABS, and PLA/ABS/SEBS-g-MA blends showed a well-defined peak at θ = 16.5° due to the diffraction of the (200) plane and a smaller peak at θ = 18.08° of the (203) plane, indicating a distinct increase in crystallinity. These are the characteristic peaks of the α phase. The addition of SEBS-g-MA decreases the definition and intensity of the peaks.

### 4.3. FTIR

Infrared spectroscopy (FTIR) was used to study any possible physical or chemical interactions between the PLA, ABS, and SEBS-g-MA groups. Additionally, infrared spectra were employed to evaluate the structural changes in the PLA, ABS, PLA/ABS, and PLA/ABS/SEBS-g-MA blends containing 5, 7.5, and 10 wt% SEBS-g-MA before and after annealing heat treatment. The main absorption bands of PLA and ABS, and the absorption bands related to SEBS-g-MA, were observed.

**Figure 5:**
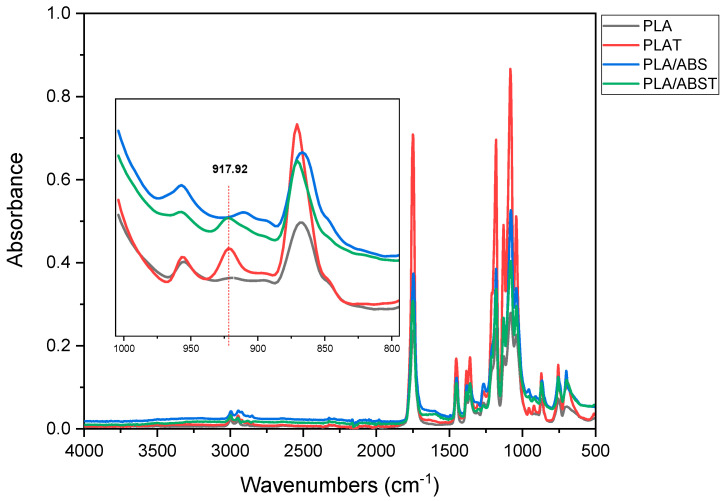
FTIR spectra showing the effect of thermal treatment on the crystallinity of PLA and the PLA/ABS blend.

Figure 5 shows the FTIR spectra of neat PLA before and after treatment. The PLA spectrum shows bands at 871 cm^−1^, referring to C-C groups, 1079, 1182, and 1267 cm^−1^, which are assigned to the stretching of the C-O bond of C-O-C, 1360, 1382, and 1453 cm^−1^, which are assigned to the bending and deformation of the C-H bond, 1750 cm^−1^, which represents the stretching of the carbonyl (C=O) of the ester group of PLA, and 2950 and 2998 cm^−1^, which are assigned to the asymmetric and symmetric stretching of the C-H bond of the CH_3_ group. Andrés et al. (2023) [40] distinguished other bands that are linked to the presence of an amorphous phase (868.6 cm^−1^) and a crystalline phase (756.6 and 701 cm^−1^). An increase in the intensity of the bands was noticed in the spectrum of the heat-treated PLA.

Figure 5 shows a detailed spectrum of PLA in the 800 to 1000 cm^−1^ range. The appearance of a band at 917.9 cm^−1^ in the heat-treated PLA is evidence that an increase in crystallinity was promoted by annealing. The band at 917.9 cm^−1^ is absent for the PLA without treatment, corroborating the XRD and impact strength data.

**Figure 6:**
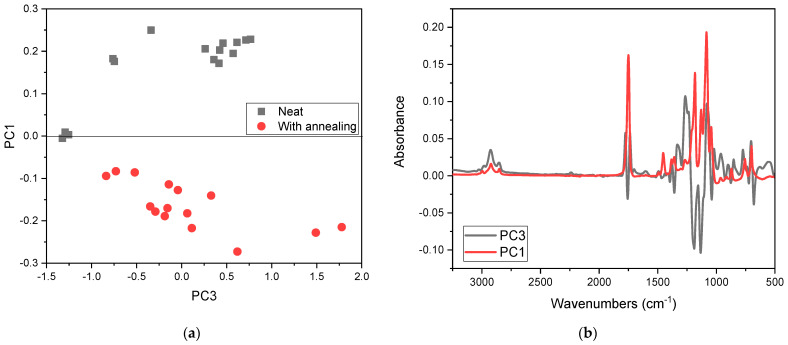
PCA scores for unannealed and annealed PLA, PLA/ABS, and PLA/ABS with 5%, 7.5%, and 10% SEBS-g-MA (**a**), and the corresponding loadings (**b**).

A main component analysis (PCA) was performed to confirm SEBS-g-MA incorporation and the effect of annealing on the PLA/ABS. This chemometric technique helps, as one of its objectives, to find the similarities between the evaluated samples and if it is possible to identify some unnoticed information, such as, in this case, the spectral data obtained by mid-infrared. Figure 6 shows the PCA scores and loadings for the samples with and without annealing. This graph represents the co-ordinates of the samples in the space of the main components (PCs).

The samples without the treatment are grouped higher in the score plot and were more influenced by bands at 1453.8, 1749.5, 755.6, and 700.2 cm^−1^ in PC1. On the other hand, the samples that underwent heat treatment are more spread out in the lower aspect in PC1, mainly due to the bands at 2981.7, 2926.2, 2854.3, 1295.1, 926.1, and 681.7 cm^−1^, which are very prominent in PC3.

**Figure 7:**
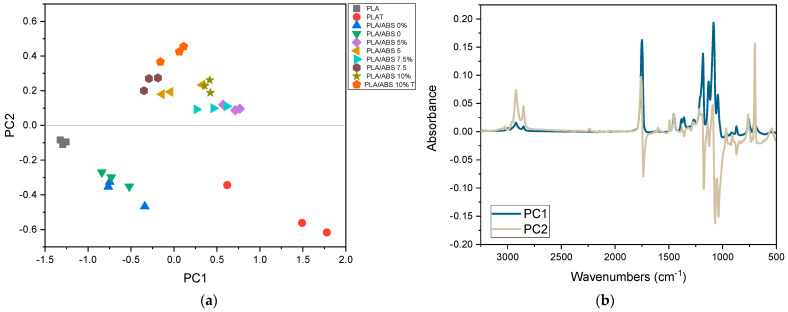
PCA scores for all PLA, PLA/ABS, and PLA/ABS/SEBS-g-MA samples with and without annealing (**a**) and the corresponding loadings (**b**).

Figure 7 shows the PCA scores and loadings for all samples as a function of compatibilization.

Figure 7 shows that PCA separated the samples into two distinct groups, indicating that SEBS-g-MA was incorporated into the PLA/ABS/SEBS-g-MA system since the compatibilized samples are more distant. The groups refer to the samples of PLA/ABS and PLA/ABS with 5% SEBS-g-MA, PLA/ABS with 7.5% SEBS-g-MA, and PLA/ABS with 10% SEBS-g-MA, respectively. The bands responsible for this classification, indicated by the PC1 weights, were: 2922.2, 2852.3, 761.8, and 700.3 cm^−1^. The band at 1757.8 cm^−1^, assigned to the carbonyl, presents a lower intensity in the compatibilized samples than the pure ones (without compatibilizer), indicating that there was an esterification reaction, i.e., the maleic anhydride of SEBS-g-MA reacts with the terminal hydroxyls of PLA and, at the same time, with the styrene blocks of ABS.

### 4.4. Mechanical Properties

**Figure 8:**
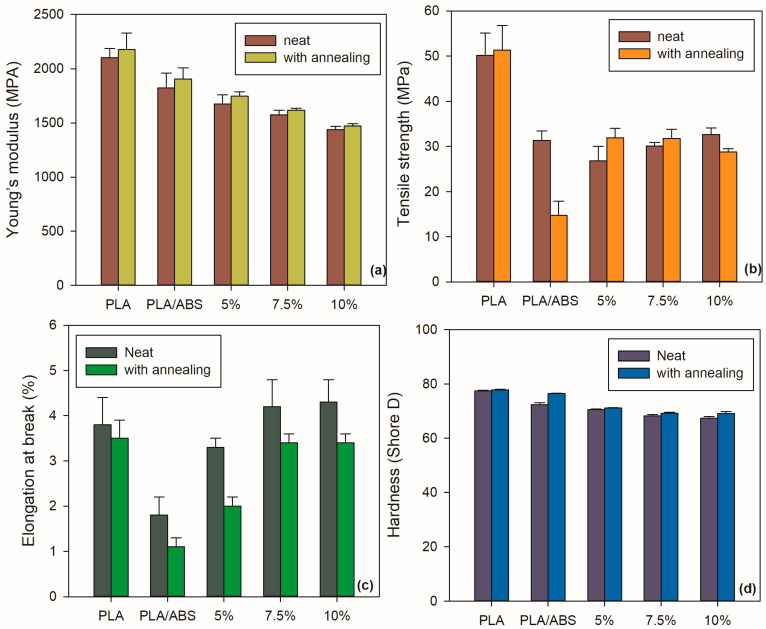
Mechanical properties of unannealed and annealed neat PLA and PLA/ABS/SEBS-g-MA blends containing different concentrations of SEBS-g-MA: (**a**) elastic modulus, (**b**) tensile strength, (**c**) impact strength, (**d**) elongation at break.

Figure 8 shows the mechanical properties of the PLA, PLA/ABS, and PLA/ABS/SEBS-g-MA systems with and without annealing. Table 3 presents the changes in tensile strength, elastic modulus, and elongation at break. PLA is a brittle polymer with a high Young’s modulus (E) of around 2.1 GPa, a tensile strength of 50.1 MPa, and a low elongation at break of around 3.8%.

According to Figure 8, the PLA/ABS blend presented significantly deteriorated mechanical properties, resulting in a brittle material (when compared to neat PLA) due to the lack of compatibility between the two polymers. By adding SEBS-g-MA, both tensile strength and elongation gradually improved when compared to the PLA/ABS blends, especially as the SEBS-g-MA content increased. The hydroxyl group at the end of the PLA macromolecule can interact with the maleic anhydride groups in SEBS-g-MA to possibly form a grafted copolymer at the interface of the blends [39]. These interactions can stabilize the interface, reducing interfacial tension and thereby increasing viscosity and interfacial adhesion. The improved adhesion explains why PLA/ABS/SEBS-g-MA exhibited higher tensile strength than the PLA/ABS blend [23]. The effect of the plasticization of SEBS-g-MA in PLA and ABS can explain the increased elongation at break.

The PLA/ABS/SEBS-g-MA blend presented an increase in elongation at break for all compositions when compared to the neat blend, i.e., the presence of SEBS contributed to improving the elongation property. The compositions exceeding 10 wt% SEBS-g-MA showed better elongational properties than the neat polymers because of the plasticizing effect of the compatibilizer in PLA and ABS.

Shore D hardness measures the resistance of the material to penetration. The neat PLA showed the highest Shore D hardness value, owing to its rigid behavior, as evidenced by its elastic modulus. The addition of 40 wt% ABS slightly reduced the Shore D hardness in the PLA/ABS system, considering that ABS presents more flexibility, which is supplied by the butadiene phase. As the SEBS-g-MA content increased, there was a tendency for a progressive reduction in the Shore D hardness of the PLA/ABS/SEBS-g-MA blends. This phenomenon can be attributed to the increase in the elastomeric fraction in the PLA/ABS/SEBS-g-MA blends since the compatibilizer is flexible. The heat treatment did not affect the Shore D hardness of PLA once the results were similar before and after the treatment. The annealing treatment was more effective in recovering the Shore D hardness of the blends, especially in the case of PLA/ABS.

### 4.5. Impact Strength

**Figure 9:**
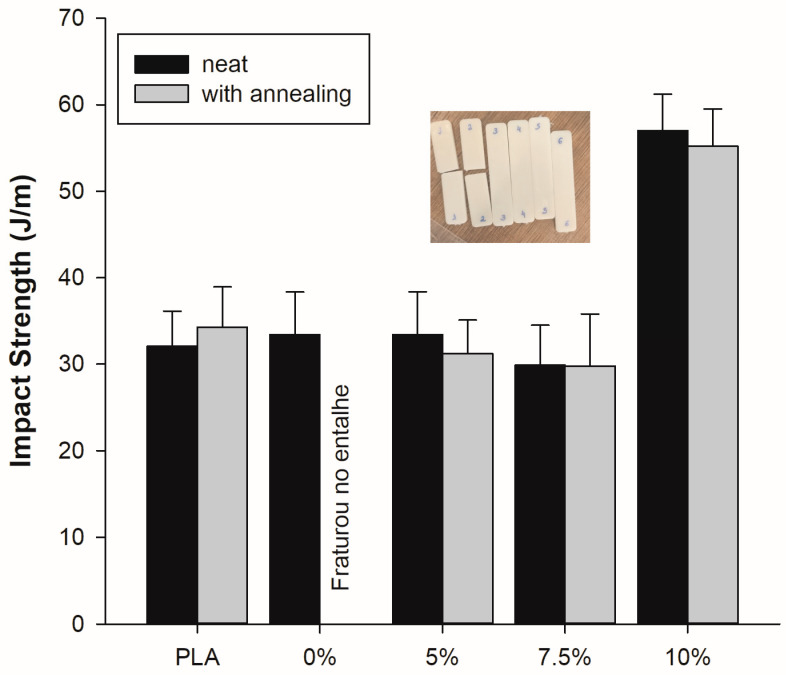
Impact strength of unannealed and annealed neat PLA and PLA/ABS/SEBS-g-MA blends containing different concentrations of SEBS-g-MA.

Figure 9 displays the impact strength data of the PLA, PLA/ABS, and PLA/ABS/SEBS-MA blends, with and without annealing. The impact strength of neat PLA was only 32.1 J/m, showing typical brittle behavior. Adding 40 wt% ABS did not improve the impact strength of PLA, as the non-compatibilized blends exhibited brittle fractures. The brittle behavior indicates that ABS could not act as an impact modifier in the PLA matrix. In addition, incorporating 5 wt% and 7.5 wt% SEBS-g-MA did not improve the toughening mechanism, given that the impact strength results are within the experimental error of PLA and the PLA/ABS blends. Only the blends compatibilized with 10 wt% SEBS-g-MA showed an improvement in impact strength. In this case, impact strength increased by 78.1% and 71.2% when compared to PLA and PLA/ABS, respectively. Therefore, to compatibilize the PLA/ABS blend with SEBS-g-MA, a critical content of at least 10 wt% must be added.

The impact strength of the PLA/ABS/SEBS-g-MA (10 wt%) blend was 57.2 J/m, almost double that of the virgin PLA/ABS blend (32.1 J/m). This result may be due to the good interfacial adhesion between the PLA matrix, ABS, and SEBS-g-MA, resulting in a material that changes from brittle to ductile. The elastomeric nature of SEBS-g-MA allowed for the dissipation of impact energy, which led to better toughness in the PLA/ABS/SEBS-g-MA blends (10 wt%). Sangeetha et al. (2016) [22] revealed that SEBS-g-MA imparted plasticizing and wetting characteristics to PLA, which improved the impact properties through toughening mechanisms, such as multiple crack formation and cavitations. This finding suggests that SEBS-g-MA may also be adding to the toughening of the PLA/ABS/SEBS-g-MA system, promoting improved impact strength results.

The heat treatment on PLA only slightly increased the impact strength. However, this was not a significant increment and fell within the margin of experimental error. The same trend was found for the PLA/ABS and PLA/ABS/SEBS-g-MA blends, suggesting that higher crystallinity (DRX) deteriorated the toughening mechanism. The PLA/ABS system revealed a high degree of embrittlement, with the samples fracturing during notching, as seen in Figure 9. Again, the PLA/ABS/SEBS-g-MA blend maintained the highest performance under impact testing, with a value of 55.2 J/m, which is comparable to that obtained before annealing. However, it is important to point out that the SEBS-g-MA compatibilizer preserved the stability of the PLA/ABS/SEBS-g-MA blends under the notch effect, considering that they did not break, as verified in the PLA/ABS sample.

### 4.6. Thermogravimetric Analysis (TGA)

**Figure 10:**
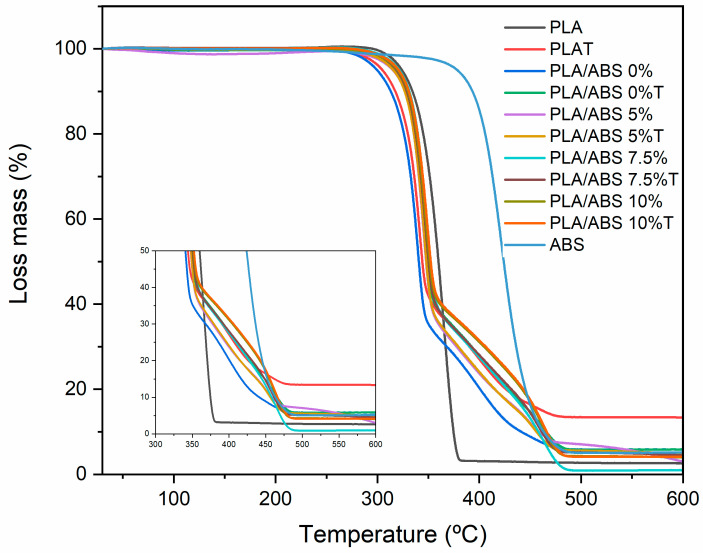
Mass vs. temperature of the neat polymers and the untreated and annealed PLA/ABS blends with different concentrations of SEBS-g-MA.

Figure 10 displays a decomposition profile of the annealed and non-annealed PLA and the PLA/ABS blends with different concentrations of SEBS-g-MA. The thermogravimetric results of the neat components and the prepared formulations can be seen in Table 4. The T_onset_, the temperature delimiting the onset of mass loss, and T_endset_, indicating the final decomposition temperature, are shown. PLA and ABS present only one step in polymer chain degradation, corroborating what has already been reported [26]. However, ABS has higher thermal stability than PLA due to the presence of acrylonitrile in its main chain, which helps to displace thermal decomposition at higher temperatures.

In the TGA profile, the neat PLA showed the lowest thermal stability. The degradation initiated at 353.38 °C and concluded at 385.28 °C. However, for the annealed neat PLA, the final decomposition temperature reached 490.8 °C, i.e., it showed thermal degradation above the untreated PLA. Hence, the thermal stability slightly increased due to more perfect crystal variants [13].

The PLA/ABS blends showed two stages of decomposition, referring to the behavior of the neat polymers. The first stage occurred between 342 and 376 °C, and the second, ascribed to ABS decomposition, occurred between 361.7 and 518.3 °C. The heat treatment shifted the decomposition range to higher temperatures when compared to the untreated blend sample.

Based on Figure 10, the PLA/ABS blends showed lower thermal stability when compared to the compatibilized blends (PLA/ABS/SEBS-g-MA) and neat PLA, which was already expected due to the weak interactions between PLA and ABS. An increase in the temperature range for stability was observed upon SEBS addition to the PLA/ABS blend. The higher the added SEBS content, the more the curves shift towards the thermal behavior of neat PLA. This curve shift indicates that SEBS-g-MA improved the interaction between the components and promoted a stabilizing effect, improving thermal stability. The literature has shown that increasing interfacial interactions promotes morphology stabilization in polymer blends and generally improves their thermal properties.

### 4.7. Differential Scanning Calorimetry (DSC)

**Figure 11:**
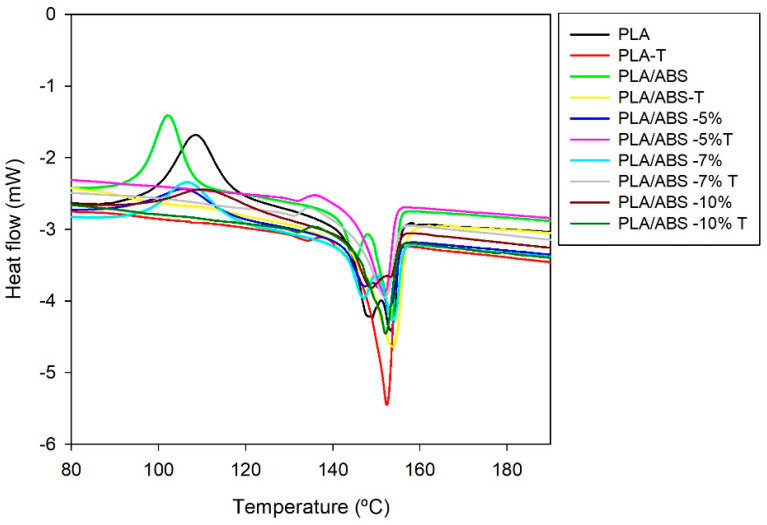
Heat flow vs. temperature during heating for neat PLA and the neat and annealed PLA/ABS blends with different concentrations of SEBS-g-MA. Curve obtained by heating up to 200 °C with a heating/cooling rate of 10 °C/min.

Figure 11 shows the DSC curves for heating. Table 5 shows the melting and crystallization parameters of PLA and its binary blends with and without the compatibilizing agent. During heating (Figure 11), the glass transition temperature (Tg) of PLA and the other systems is related to transitions in the 55–59 °C range. It can be observed that the neat PLA presented the exothermic peak related to the cold crystallization temperature, which indicates that the crystallization process of PLA was not complete after processing and that SEBS-g-MA influenced the intensity of the peaks. The double peak presented by PLA, around 149.1 and 153.3 °C, is characteristic of the melting of the β and α phases [26].

The blends’ melting (Tm) temperature showed no significant differences when compared to neat PLA. The addition of SEBS-g-MA affects PLA by reducing crystallinity. However, the degree of crystallinity of the blends increased with annealing, as expected. On the other hand, it is possible to observe the disappearance of the crystallinity peak for the blends with annealing in Figure 11. The interactions between PLA, ABS, and SEBS-g-MA may hinder the ordering mechanism, i.e., the compatibilizing agent reduces the melting enthalpy (ΔHf) due to the reduction in crystallinity. It is worth mentioning (from Figure 7 and Table 4) that, after annealing, there was an increase in crystallinity, corroborating the XRD data. The PLA/ABS/SEBS-g-MA blends showed different behavior during reheating, with the appearance of two shallow peaks attributed to the melting of the ethylene groups of ABS. Rigoussen et al. (2019) [17] connected this reduction in crystallinity to the difficulty of crystallization in PLA in the presence of a second dispersed phase. The second phase makes the arrangement of PLA chains difficult, reducing its crystallinity. The melting and glass transition temperature of the PLA did not show any significant changes.

### 4.8. HDT

**Figure 12:**
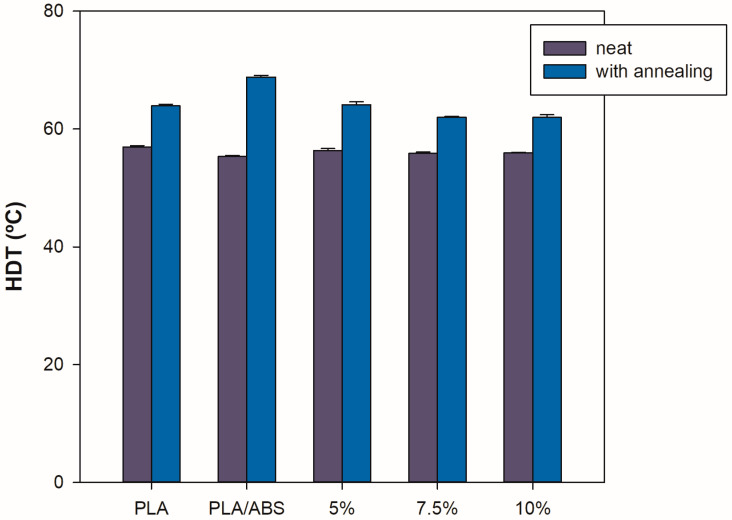
Heat deflection temperature of unannealed and annealed PLA and PLA/ABS/SEBS-g-MA blends containing different concentrations of SEBS-g-MA.

Figure 12 shows heat deflection temperature (HDT) behavior as a function of composition regarding the PLA/ABS blends with 0, 5, 7.5, and 10 wt% SEBS-g-MA and the heat treatment performed. The HDT of the neat PLA was in the range of 58 °C. The incorporation of ABS as an impact modifier promoted a slight decrease in the thermal deflection temperature of the blends. Although ABS presents a higher HDT value than PLA, the continuous phase (PLA matrix) contributes more significantly to HDT than ABS, explaining why the HDT values are close to neat PLA. The blends compatibilized with different SEBS-g-MA contents showed no meaningful difference between them. A little increment was observed when compared to the neat blends and the neat PLA, contributing to sustaining the thermal deflection temperature (HDT) levels. The heat-treated compositions presented higher HDT results than the neat PLA and their respective untreated blends. This increase reflects the increase in the degree of crystallinity promoted by annealing, as confirmed in the XRD patterns. The heat treatment was more effective for the blend without compatibilization. However, the results presented are relevant from a technological perspective, considering the achievement of some of the tailoring of the properties. The PLA/ABS/SEBS-g-MA (10 wt%) blends after annealing presented a high impact strength and, at the same time, an increased HDT. These improvements suggest that this composition was ideal for improving the main shortcomings of PLA, i.e., low impact strength and HDT.

### 4.9. MEV

**Figure 13:**
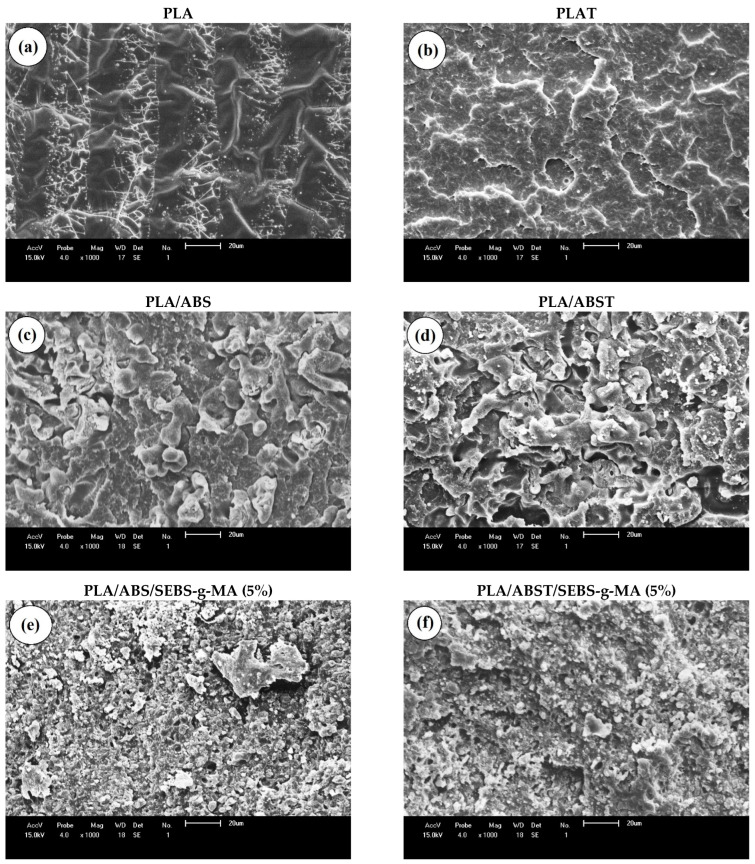

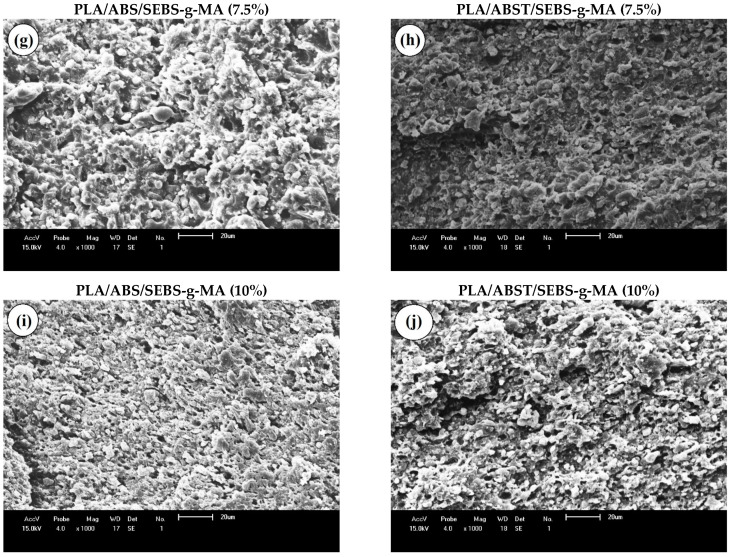
SEM images for the compositions at 1000× magnification.

Figure 13 shows the analyses of the fracture surface of PLA and polymer blends, with and without annealing heat treatment, at 1000× magnification. Figure 13a,c show that the annealing heat treatment modified the fracture surface, as PLA showed undulations with smooth and deformed regions, while PLAT had a more homogeneous fracture. Although the surface of PLA and PLA after annealing (PLAT) saw a variation in fracture behavior, there were no significant changes in impact strength. Figure 13c–j show phase separation in the polymer blends, wherein the ABS particles are dispersed in the PLA matrix, evidencing immiscible mixtures. The PLA/ABS before and after annealing and the PLA/ABST blends exhibited a rather coarse fracture surface with large and elongated particles, suggesting coalescence behavior [41,42]. However, the deformation mechanism of the PLA/ABS blends was more stable during impact testing than that of PLA/ABST, leading to higher impact strength, as shown in Figure 9. The heat treatment in the PLA/ABST system produced a more unstable morphology with a marked level of voids, which may have generated low impact strength. This behavior is probably associated with the combination of PLA crystallization and the large average size of the ABS dispersed phase, producing high brittleness.

Figure 13e–j represent the fracture surfaces of the PLA/ABS blends compatibilized with 5, 7.5, and 10 wt% SEBS-g-MA, respectively. There was a reduction in ABS particle size in the PLA matrix, indicating that SEBS-g-MA promoted chemical interactions. The refining of the particle size possibly indicates a polyesterification reaction between the hydroxyl terminal group of PLA and the maleic anhydride group of SEBS-g-MA, which has also been demonstrated in the literature [43]. At the same time, the styrene group of SEBS-g-MA can dissolve in the ABS phase, generating morphology refinement, as verified in the PLA/ABS/SEBS-g-MA samples. In the literature [44,45], it was reported that the compatibilizer diffuses to the interface between the phases of the polymer blends, promoting a reduction in interfacial energy and preventing the coalescence of particles. However, a fraction of the compatibilizer can also remain dispersed in the PLA matrix, contributing to toughening. Although SEBS-g-MA promoted the formation of more refined morphology, some pulled-out and large particles still suggest partial compatibilization. Apparently, the incorporation of 5 wt% and 7.5 wt% SEBS-g-MA into the PLA/ABS mixture did not affect morphology since the fracture surfaces are similar. When adding 10 wt% SEBS-g-MA, the fracture showed a higher level of plastic deformation, indicating higher resistance to crack propagation. This observation suggests that a higher concentration of SEBS-g-MA increased the interaction and enhanced the toughening mechanism, causing higher performance under impact. In terms of the effect of annealing heat treatment, regardless of the SEBS-g-MA content, no significant changes in the morphology of the PLA/ABS/SEBS-g-MA blends were observed, explaining the similar impact strength properties.

## 5. Conclusions

The present work used an extrusion method to obtain SEBS-g-MA-compatibilized PLA/ABS blends. The effect of annealing to anticipate secondary crystallization was also evaluated. These methods proved effective in making PLA more competitive by improving some of the properties that limit its application. The torque rheometry results show a reduction in degradation rate with increasing SEBS-g-MA content in the matrix, indicating that the blends compatibilized with 10 wt% SEBS degrade much less than the neat PLA and ABS. The values of the elongation at break of the blends showed an increase with the addition of SEBS-g-MA at levels of 7.5% and 10%, which indicates an increase in ductility. The percentage of 10 wt% SEBS-g-MA was ideal for improving impact strength, leading to an increase of 57.19 J/m (45%), which is almost double that of the pristine blends. The SEM images proved that for the systems with a higher percentage of SEBS-g-MA, there was higher compatibility once the refinement of the morphology and a higher interaction at the interface was observed. The FTIR spectra of the blends were very similar to the algebraic sum of the spectra of the neat components, which corresponds to a completely immiscible blend. However, adding the compatibilizer agent promoted esterification reactions, in which the maleic anhydride group reacted with the terminal hydroxyls of the PLA and, simultaneously, with the styrene blocks of the ABS. The thermogravimetric analysis indicated that the thermal stability of PLA/ABS improved with the addition of SEBS-g-MA, and the crystallization of PLA was prevented. Regarding annealing, there was also a remarkable improvement in the heat deflection temperature in the systems subjected to annealing when compared to those that were not. The XRD and FTIR results proved that annealing increased the crystallinity of the sample; consequently, a decrease in mechanical properties, such as tensile strength and impact strength, was observed.

## Figures and Tables

**Table 1:** Compositions of the PLA/ABS and PLA/ABS/SEBS-MA blends.

Designation	Composition		
	(%) PLA	(%) ABS	(%) SEBS-g-MA
PLA	60	0	0
PLA/ABS	60	40	0
PLA/ABS-5%SEBS-g-MA	60	35	5
PLA/ABS-7.5%SEBS-g-MA	60	32.5	7.5
PLA/ABS-10%SEBS-g-MA	60	30	10

**Table 2:** Parameters obtained during processing.

Systems	Processing Parameters				
	$\bar{T}$ (°C)	*Z* (Nm)	$\overset{¯}{Z^{*}}$ (Nm)	$dZ^{*}/dt$ (Nm/min)	*R_Z_* (%/min)
ABS	205.0 ± 0.01	38.2 ± 0.02	48.6 ± 0.05	−2.26 ± 0.75	4.6 ± 0.02
PLA	200.2 ± 0.01	4.3 ± 0.03	4.34 ± 0.03	−1.58 ± 0.54	36.2 ± 0.12
PLA/ABS	200.7 ± 0.01	2.6 ± 0.03	2.7 ± 0.04	−1.77 ± 0.59	65.1 ± 0.21
5% SEBS	202.1 ± 0.01	9.1 ± 0.05	10.0 ± 0.06	−2.64 ± 0.87	26.3 ± 0.09
7.5% SEBS	202.2 ± 0.01	13.0 ± 0.02	14.5 ± 0.02	−0.82 ± 0.87	5.7 ± 0.14
10% SEBS	203.1 ± 0.08	13.7 ± 0.02	15.8 ± 0.02	−0.69 ± 0.45	4.3 ± 0.05

**Table 3:** Mechanical properties of PLA, ABS, and PLA/ABS blends with or without annealing containing different concentrations of SEBS-g-MA.

		Mechanical Properties				
	Designation	Impact Strength (J/m)	Young’s Modulus (MPa)	Tensile Strength (MPa)	Elongation at Break (%)	Hardness Shore D
Neat	PLA	32.1 ± 4.05	2101.4 ± 85.70	50.2 ± 4.90	3.8 ± 0.60	77.5 ± 0.15
	PLA/ABS-0%	33.4 ± 4.94	1823.8 ± 137.10	26.8 ± 3.20	1.8 ± 0.40	72.4 ± 0.69
	PLA/ABS-5%	33.4 ± 4.94	1675.0 ± 83.40	31.9 ± 2.10	3.3 ± 0.20	70.5 ± 0.22
	PLA/ABS-7.5%	29.9 ± 4.61	1574.6 ± 43.60	32.6 ± 1.50	4.2 ± 0.60	68.2 ± 0.46
	PLA/ABS-10%	57.2 ± 4.22	1437.0 ± 30.90	30.1 ± 0.80	4.3 ± 0.50	67.4 ± 0.60
With annealing	PLA	34.2 ± 4.67	2176.4 ± 152.80	51.3 ± 5.50	3.5 ± 0.40	77.8 ± 0.25
	PLA/ABS-0%	-	1903.2 ± 40.10	14.7 ± 3.20	1.1 ± 0.20	76.5 ± 0.15
	PLA/ABS-5%	31.2 ± 3.91	1747.4 ± 40.10	31.3 ± 2.10	2.0 ± 0.20	71.1 ± 0.18
	PLA/ABS-7.5%	29.8 ± 6.01	1615.6 ± 19.20	31.8 ± 2.00	3.4 ± 0.20	69.2 ± 0.40
	PLA/ABS-10%	55.2 ± 4.30	1471.6 ± 19.20	28.8 ± 0.70	3.4 ± 0.20	69.1 ± 0.64

**Table 4:** Results obtained from the TGA curves.

Systems		T_onset_ (°C)	T_endset_ (°C)
Neat	PLA	342.9	385.2
	PLA/ABS-0%	328.1	518.3
	PLA/ABS-5%	333.1	513.9
	PLA/ABS-7.5%	334.4	520.0
	PLA/ABS-10%	332.9	503.6
With annealing	PLA T	317.5	490.8
	PLA/ABS T	333.1	505.8
	PLA/ABS-5%T	331.3	489.1
	PLA/ABS-7.5%T	332.4	499.6
	PLA/ABS-10%T	334.5	497.1

**Table 5:** Thermal properties during the heating of unannealed and annealed PLA, ABS, and PLA/ABS blends containing different concentrations of SEBS-g-MA. _(1)_ first peak; _(2)_ second peak.

Systems		Melting and Crystallization Parameters					
		tg	T_c_ (°C)	T_m_ (°C)	DH_c_ (J/g)	DH_m_ (J/g)	Xc (%)
neat	PLA	57.4.	108.5	149.1 153.3	23.8	33.5	28.7
	PLA/ABS-0%	56.4	102.2	146.3 _(1)_ 153.5 _(2)_	39.6	45.7	38.8
	PLA/ABS-5%	59.2	105.9	149.8 _(1)_ 152.8 _(2)_	29.9	37.9	40.4
	PLA/ABS-7.5%	59.1	106.7	147.1 _(1)_ 153.8 _(2)_	34.6	41.1	43.9
	PLA/ABS-10%	59.4	110.5	152.2	18.5	37.1	39.5
With annealing	PLA T	59.6	-	150.8	-	28.7	30.7
	PLA/ABS T	59.7	-	153.7	-	30.8	40.3
	PLA/ABS-5%T	58.5	-	151.8	-	42.2	45.1
	PLA/ABS-7.5%T	56.8	-	150.2	-	42.1	44.9
	PLA/ABS-10%T	58.3	-	152.2	-	37.9	39.9

## Data Availability

Not applicable.
